# Joint modelling of repeated measurements and time-to-event outcomes: flexible model specification and exact likelihood inference

**DOI:** 10.1111/rssb.12060

**Published:** 2014-04-08

**Authors:** Jessica Barrett, Peter Diggle, Robin Henderson, David Taylor-Robinson

**Affiliations:** University of CambridgeUK; Lancaster UniversityUK; University of NewcastleUK; University of LiverpoolUK

**Keywords:** Cystic fibrosis, Dropout, Joint modelling, Repeated measurements, Skew normal distribution, Survival analysis

## Abstract

Random effects or shared parameter models are commonly advocated for the analysis of combined repeated measurement and event history data, including dropout from longitudinal trials. Their use in practical applications has generally been limited by computational cost and complexity, meaning that only simple special cases can be fitted by using readily available software. We propose a new approach that exploits recent distributional results for the extended skew normal family to allow exact likelihood inference for a flexible class of random-effects models. The method uses a discretization of the timescale for the time-to-event outcome, which is often unavoidable in any case when events correspond to dropout. We place no restriction on the times at which repeated measurements are made. An analysis of repeated lung function measurements in a cystic fibrosis cohort is used to illustrate the method.

## 1. Introduction

There is a close relationship between modelling longitudinal data subject to dropout and modelling survival time data in the presence of imprecisely observed time varying covariates. In both cases we have a vector of repeated measurements *Y* and a time to event *S*. In the survival context *S* would normally be considered to be measured in continuous time, though possibly right censored. In the dropout context *S* usually corresponds to a discrete interval between scheduled measurement times. Typically the occurrence of the event terminates observation of the repeated measurements.

A common approach for both the survival time and the dropout problems is to assume conditional independence between *Y* and *S* given underlying random effects *U*. An important early reference is Wulfsohn and Tsiatis (1997), who assumed that *Y* was linear in a Gaussian random effect *U* and took a proportional hazards model for *S*, conditional on *U*. Tsiatis and Davidian (2004) have given an excellent review of work in the area to that date, and Albert and Follmann (2009) and Diggle *et al*. (2009) have given additional references including later developments. More recent contributions include Geskus (2012), Gueorguieva *et al*. (2012), Proust-Lima *et al*. (2012) and Rizopoulos (2011, 2012).

The random-effects or shared parameter approach to jointly modelling repeated measurement and event time data is conceptually attractive in many settings, but its routine application is hampered by computational difficulties. Fast but approximate methods have been developed for some forms of joint model (see for example Rizopoulos (2012) and references therein) but implementation remains difficult unless the random-effects component is of low dimension. Wulfsohn and Tsiatis (1997), for instance, assumed that *U* was bivariate Gaussian and adopted a Laird and Ware (1982) random intercept and slope model for the longitudinal trajectory. Henderson *et al*. (2000) argued that when follow-up is relatively long then it is unreasonable to assume a sustained trend in the trajectory of *Y* and advocated inclusion of an unobserved stationary Gaussian process *W*(*t*) in a linear predictor for *Y* to bring more flexibility. In principle this assumes the presence of an infinite dimensional random effect but, under either a discrete dropout or a semiparametric proportional hazards model for *S*, likelihood inference requires the value of *W*(*t*) at only measurement times or event times. Hence *W*(*t*) can be represented by a finite dimensional vector *U*. The dimension increases with sample size and there is no generally available software for this type of model.

In this paper we propose an approach which admits exact likelihood inference for a wide range of random-effect specifications. The key is to consider events *S* to occur only at a discrete set of potential times. In principle, the discretization can be made arbitrarily fine, but at the expense of increasing computational effort. Hence, the practical advantage of our approach relies on its remaining computationally feasible for a discretization that is sufficiently fine to capture the essential features of its continuous time limit. When the events correspond to dropout, their recorded times of occurrence are typically confined to a discrete set of scheduled measurement times. When the event is a survival time then our approach is a form of coarsening at random (Heitjan and Rubin, 1991). For example, in Application: disease progression and survival in cystic fibrosis patients we describe an example involving survival in a cystic fibrosis cohort, where we choose to measure survival by calendar year of death. With 10 years of follow-up and no evidence of significant local variation in hazard rates this gives adequate granularity. There is no need for the timescale to be discretized in the submodel for the repeated measurements *Y* under our approach, which therefore allows for irregular and subject-specific measurement timings.

The general model and some special cases are set out in Section Model. In Section Inference we derive the key likelihood-based methods on the basis of recent work on the extended skew normal family of distributions. Simulations to assess the performance of the method are described in Section Simulation studies. An examination of efficiency is presented in Section Efficiency under coarsening at random and in Section Application: disease progression and survival in cystic fibrosis patients we describe application to the cystic fibrosis cohort study. Concluding discussion is presented in Section Discussion.

The programs that were used to analyse the data can be obtained from

http://wileyonlinelibrary.com/journal/rss-datasets

## 2. Model

We take a shared parameter approach, with the common assumption that event times *S* and longitudinal data *Y* are independent conditionally on random effects *U*.

### 2.1. General formulation

We assume that subject *i* provides repeated measurements *Y*_*i*_={*Y*_*ij*_:*i*=1,2,…,*N*;*j*=1,…,*n*_*i*_} at follow-up times *t*_*ij*_, together with a time-to-event outcome *S*_*i*_, which terminates the observation of *Y*. Time varying covariates are allowed. We write *x*_*ij*_ for covariates that are operative on *Y*_*ij*_, and 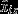 for covariates that are operative at time *s* on the event process. For the remainder of this section we drop the subscript *i* and consider a generic subject. It will be implicit that the number and timing of measurements can vary between subjects.

We assume two timescales: a discrete timescale for events, which without loss of generality we can label as 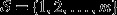, and a continuous timescale 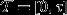 for measurements. We also assume that there is a surjection *s*(*t*) from 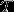 to 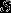. For instance, 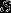 might be a partition of 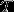. We consider 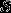 to represent a series of time intervals; for example if the timescale 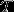 is years then 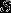 might contain yearly or 6-monthly intervals. Alternatively, 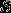 might contain time intervals of different lengths, or it might represent intervals between scheduled measurement times. We denote by t^*^(*s*) the midpoint of the set of times in 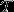 that map to 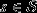.

#### 2.1.1. Random effects

We model the random effects as a *p*-vector *U*=(*U*_1_,…,*U*_*p*_)^T^, assuming a Gaussian distribution *U*∼*N*(0,Σ) with a general covariance structure.

#### 2.1.2. Event times

With the skew normal results in mind, we adopt a probit model for the discrete hazard function (equivalently the dropout model), which is very similar to the more widely used logistic model. For the most general model we define *W*(*s*)=*B*(*s*)*U* to be a 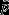-vector of linear combinations of the random effects *U*, where *B*(*s*) is a 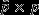 matrix which may depend on the time interval *s*. Then we assume that

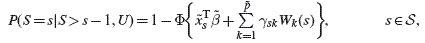
1where Φ(·) is the standard normal distribution function. In equation 1 the survival model is allowed to depend on time through covariates that are contained in a *p*_1_-vector 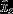. In principle a non-linear function of time could be used, and often a separate intercept would be used for each time interval. In our examples we shall focus on models for which the probit probability of surviving a time interval is linearly dependent on time. The association between survival and the random effects can also vary with time, although all examples in this paper will assume that *γ*_*sk*_ does not depend on *s*.

Note that the model thus defined is a sequential probit model because the probability of surviving a time interval is conditional on having survived all previous time intervals. Albert and Chib (2001) have provided discussion on the application of sequential models in survival.

#### 2.1.3. Repeated measurements

We consider a linear mixed effects Gaussian model for the sequence of repeated measurements, *Y*={*Y*_*j*_:*j*=1,…,*n*}, at measurement times *t*_*j*_. At time *t*_*j*_ we assume

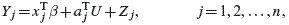
2where *x*_*j*_ and *a*_*j*_ are a *p*_2_-vector and a *p*-vector respectively, of possibly time-dependent covariates. The {*Z*_*j*_} are mutually independent measurement errors, taken as *Z*∼MVN(0,*ν*^2^*I*) with *I* an identity matrix. We can write expression (2) in the vector form

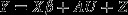
3where *X*=(*x*_1_,*x*_2_,…,*x*_*n*_)^T^ and *A*=(*a*_1_,*a*_2_,…,*a*_*n*_)^T^.

### 2.2. Examples

#### 2.2.2. Random intercept

Our formulation includes the random-intercept model. Assume that *U* is a scalar random effect with *N*(0,*σ*^2^) distribution. Then set *a*_*j*_=1 for all *j* and *W*_*s*_=*U*, with *γ*_*s*_=*γ* for all 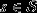.

#### 2.2.3. Random intercept and slope

Let (*U*_1_,*U*_2_)^T^ be zero mean bivariate normal with variance matrix

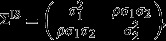
and set *a*_*j*_=(1,*t*_*j*_)^T^. For 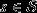 set *W*(*s*)=*U*_1_+*U*_2_ t^*^(*s*) and *γ*_*s*_=*γ* for all 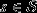. Thus we can model a sustained trend in the survival hazard, albeit with a piecewise constant form. Given that we work with events in discrete time this is unavoidable. Alternatively we can adjust the model to allow the intercept and slope random effects to enter the event times model directly, setting *W*(*s*)=(*U*_1_,*U*_2_)^T^ and *γ*_*s*_=(*γ*_1_,*γ*_2_)^T^.

#### 2.2.3. Stationary Gaussian process

For the stationary Gaussian process model we assume that *U* is an *m*-vector with variance matrix

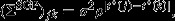
so that the random effects correspond to a discretely observed stationary Gaussian process. Here *W*(*s*)=*U*_*s*_ and *γ*_*s*_=*γ* for all 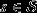. We assume that measurements *Y*_*j*_ and *Y*_*k*_ with *s*(*t*_*j*_)=*s*(*t*_*k*_) share the same *U*_*s*_. We define *a*_*j*_ to be an *m*-vector with value 1 at element *s*(*t*_*j*_) and 0 elsewhere. If fluctuation at a very short timescale is thought to be materially important then a fine discretization 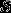 is needed to capture this behaviour. Note that the discretization does not need to scale with sample size. By extending *W*(*s*) we can allow survival or dropout to depend on the prior history of random effects; for example for one time lag set *W*(*s*)=(*U*_*s*_,*U*_*s*−1_)^T^ and *γ*_*s*_=(*γ*,*γ*_lag_)^T^.

#### 2.2.4. Stationary Gaussian process plus random intercept and slope

We can combine a stationary Gaussian process with a random intercept and slope by defining *U* to be an (*m*+2)-vector with variance matrix

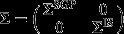
Now *W*(*s*)=(*U*_*s*_,*U*_1_,*U*_2_) and *γ*_*s*_=(*γ*,*γ*_1_,*γ*_2_) for all *s*>1.

## 3. Inference

We now set out the general form of the likelihood for the class of models that was defined in Section General formulation. For simplicity we shall continue with the contribution of a single individual only.

We shall make use of results concerning the properties of the skew normal distribution ( Azzalini, [Bibr b6],[Bibr b7]; Arnold and Beaver, 2000; Arnold, 2009) to obtain a closed form for the likelihood. In particular, a multivariate Gaussian hidden truncation distribution that was considered by Arnold (2009) leads to the result

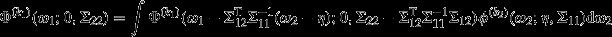
4where *ω*_1_, *ω*_2_ and *η* are *k*_1_×1, *k*_2_×1 and *k*_2_×1 vectors respectively, and Σ_11_, Σ_22_ and Σ_12_ are concordant variance and covariance matrices. Here *ϕ*^(*k*)^(*x*;*μ*,Σ) is the *k*-dimensional Gaussian density with mean vector *μ* and covariance matrix Σ and Φ^(*k*)^(*x*;*μ*,Σ) is the corresponding distribution function. By rearranging the likelihood so that it takes the form of the integrand on the right-hand side of equation 7, we can use this result to integrate out the random effects to arrive at a closed form expression.

### 3.1. Preliminaries

We begin by rearranging the longitudinal and random-effects components of the likelihood so that they take the form of the Gaussian density function under the integrand in equation 7.

Assume that the dropout or survival time is *s* and introduce an indicator *δ* of an event being observed (*δ*=1) or right censoring (*δ*=0). The random-effects and longitudinal components of the likelihood are

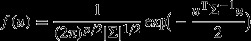
and

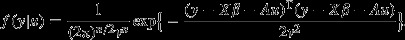
Note that *f*(*y*|*u*) *f*(*u*) contains two terms under the exponential which are quadratic in *u*. We can complete the square in *u* so that *u* appears in a single quadratic term (see Appendix A for details). It will be convenient to collect all unknown parameters into vector *θ* and to write

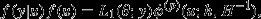
5where

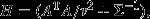


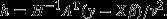
Note that *ϕ*^(*p*)^(*u*;*h*,*H*^−1^) depends on a subvector of the parameter vector *θ* through *h* and *H*. The expression for *L*_1_(*θ*;*y*) is

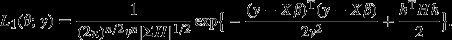


Turning to the event times, we have

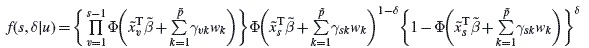
6where *w*=*B*(*s*)*u* and Φ(·) is the standard Gaussian cumulative distribution function. Now define 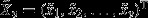, and an 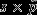 matrix Γ such that Γ_*vk*_=*γ*_*vk*_. Then equation 14 can be written as

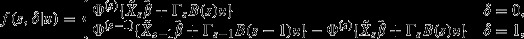
7where Γ_*s*_ denotes the first *s* rows of Γ. Here Φ^(*s*)^(·)=Φ^(*s*)^(·;0,*I*) is the standard *s*-dimensional multivariate Gaussian cumulative distribution function.

### 3.2. Likelihood

Combining expressions (5) and (7) and integrating out the random effects leads to the likelihood

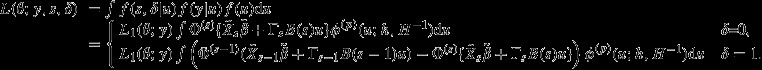
8

We can convert the right-hand side of expression 4 to the form of the integrals in expression (8) by defining the parameters *ω*_1_, *ω*_2_, *η*, Σ_11_, Σ_12_ and Σ_22_ appropriately (see Appendix A for details). Using expression (4) to obtain closed form expressions for the integrals then gives

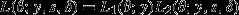
9where

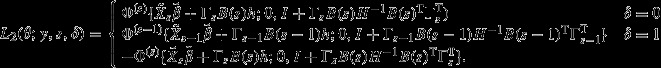
where Numerical evaluation of equation 9 is now straightforward by using available software to calculate multivariate normal probabilities; we used the R package mnormt (Azzalini, 2013). Parameter estimation can be conducted by numerical maximization of the likelihood, and candidate models can be compared by exact likelihoods or information criteria. Having specified a fully parametric model, the maximum likelihood estimator 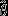 is asymptotically Gaussian, centred at the true value *θ*_0_, and with variance given by the inverse Fisher information. Standard regularity conditions apply, with some supplementation to ensure identifiability of parameters when data can be missing. Details are provided in Appendix B.

## 4. Simulation studies

We simulated repeated measurements with dropout data from the models specified by equations 1 and 2. Random effects were generated either as a stationary Gaussian process or as a random intercept and slope. Longitudinal measurements took place at *n* measurement times randomly distributed over *m* time intervals, so that individuals could have a varying number of visits in each time interval. A uniform distribution was chosen for the measurement times, motivated by our cystic fibrosis application. We assumed that dropout could occur during any time interval. For the stationary Gaussian process models we generated *m*-dimensional random effects *U*=(*U*_1_,…,*U*_*m*_)^T^ and Gaussian repeated measurements

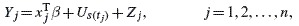
where *t*_*j*_ are the measurement times, and

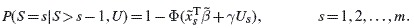
For the random intercept and slope simulations we generated bivariate random effects *U*=(*U*_1_,*U*_2_)^T^ and Gaussian repeated measurements

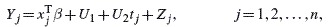
and

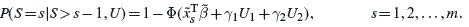


For each simulation study 500 data sets were generated; each of 1000 individuals followed over *m*=5 time intervals, with 1–10 repeated measurements per person. Survival parameters were chosen such that the death rate per time interval was 1–2%, which is similar to that observed in the cystic fibrosis application. Censoring took place at the end of the fifth time interval. Covariates that were included in both models were age, with initial values generated uniformly from the interval (15,30), and a binary covariate, which was either 0 or 1 with probability 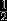. These covariates were similar to the observed covariate structure in the cystic fibrosis data.

Table1 summarizes the results of two studies with stationary Gaussian process random effects. For the first simulation (SGP I), the repeated measures and random effects were taken to have large standard deviations, similar to those observed for the cystic fibrosis patients. The simulation was repeated (SGP II) with smaller standard deviations. For both scenarios the means of the parameter estimates are close to the true values. For simulation SGP I the standard deviations of parameter estimates are relatively large for the longitudinal parameters owing to the high level of measurement error. For simulation SGP II the corresponding standard deviations are smaller, but the standard deviation of *γ* is larger. This is because the variance of the random effects is smaller, so we are effectively regressing on a covariate with a smaller range of values. Coverage probabilities for both simulation SGP I and simulation SGP II are good, with all empirical values within simulation noise of the nominal level and no consistent overestimation or underestimation of the coverage.

**Table 1:** Simulation results from a stationary Gaussian process model†

*Parameter*	*Results for simulation SGP I*				*Results for simulation SGP II*			
	*True*	*Mean*	*Coverage*	*MSE*	*True*	*Mean*	*Coverage*	*MSE*
	*value*				*value*			
*Longitudinal*								
Intercept	90	89.947	0.959	6.521	90	90.005	0.944	0.016
		(2.556)				(0.127)		
Time	−1.7	−1.700	0.941	0.074	−1.7	−1.700	0.944	0.000
		(0.272)				(0.016)		
Age at *t*_0_	−1.7	−1.696	0.957	0.013	−1.7	−1.700	0.942	0.000
		(0.115)				(0.006)		
Sex (males)	2	1.943	0.943	1.803	2	2.001	0.948	0.004
		(1.343)				(0.061)		
*Survival*								
Intercept	2	1.998	0.949	0.044	2	2.013	0.942	0.046
		(0.210)				(0.214)		
Time	0.01	0.009	0.952	0.001	0.01	0.009	0.948	0.001
		(0.031)				(0.036)		
Age at *t*_0_	0.01	0.011	0.949	0.000	0.01	0.010	0.946	0.000
		(0.009)				(0.009)		
Sex (males)	0.1	0.098	0.957	0.010	0.1	0.097	0.952	0.011
		(0.099)				(0.105)		
*γ*	0.05	0.050	0.961	0.000	0.05	0.049	0.966	0.005
		(0.003)				(0.072)		
*Others*								
*ν*	7	7.005	0.959	0.011	1	1.000	0.944	0.000
		(0.104)				(0.014)		
*σ*_*u*_	25	24.985	0.959	0.203	1	0.999	0.948	0.001
		(0.450)				(0.025)		
*ρ*	0.7	0.699	0.947	0.000	0.7	0.700	0.946	0.001
		(0.015)				(0.025)		

† Sample size 1000 and 500 replicates. Shown are the mean (with standard deviations in parentheses) parameter estimates, empirical coverage probabilities of nominal 95% confidence intervals and mean-squared errors MSE.

Table2 shows the results of two simulation studies with random intercepts and slopes. Again, for one study (IS I) data were generated with large measurement error, very high variance in the random intercept *U*_1_ and high variance for the random slope *U*_2_, as observed for cystic fibrosis patients. For the second simulation study (IS II) the variance parameters were reduced. Means of parameter estimates are once more close to the true values. The standard deviations of longitudinal parameter estimates are large for simulation IS I and small for IS II, as expected, whereas the standard deviation of estimates of *γ*_1_ is much higher for simulation IS II than for IS I. Interestingly, the standard deviation for *γ*_2_ is approximately the same in both scenarios. Coverage probabilities are once more close to the nominal levels.

**Table 2:** Simulation results from a random intercept and slope model†

*Parameter*	*Results for simulation IS I*				*Results for simulation IS II*			
	*True*	*Mean*	*Coverage*	*MSE*	*True*	*Mean*	*Coverage*	*MSE*
	*value*				*value*			
*Longitudinal*								
Intercept	90	89.842	0.940	7.743	90	89.994	0.950	0.020
		(2.781)				(0.141)		
Time	−1.7	−1.701	0.950	0.012	−1.7	−1.701	0.952	0.001
		(0.109)				(0.036)		
Age at *t*_0_	−1.7	−1.693	0.956	0.016	−1.7	−1.700	0.950	0.000
		(0.125)				(0.006)		
Sex (males)	2	2.056	0.952	1.840	2	2.003	0.956	0.005
		(1.357)				(0.071)		
*Survival*								
Intercept	2	2.051	0.946	0.054	2	2.015	0.962	0.035
		(0.226)				(0.187)		
Time	0.01	0.009	0.956	0.001	0.01	0.010	0.946	0.001
		(0.037)				(0.037)		
Age at *t*_0_	0.01	0.010	0.932	0.000	0.01	0.010	0.948	0.000
		(0.010)				(0.009)		
Sex (males)	0.1	0.112	0.966	0.010	0.1	0.113	0.938	0.012
		(0.100)				(0.107)		
*γ*_1_	0.01	0.011	0.970	0.000	0.01	0.019	0.950	0.011
		(0.005)				(0.107)		
*γ*_2_	0.1	0.112	0.970	0.011	0.1	0.107	0.946	0.007
		(0.106)				(0.084)		
*Others*								
*ν*	7	6.997	0.952	0.006	1	1.000	0.972	0.000
		(0.080)				(0.011)		
*σ*_1_	25	24.993	0.948	0.409	1	0.994	0.980	0.002
		(0.640)				(0.042)		
*σ*_2_	2	1.989	0.940	0.012	1	0.999	0.952	0.001
		(0.112)				(0.027)		
*ρ*	−0.6	−0.600	0.950	0.001	−0.6	−0.599	0.958	0.001
		(0.038)				(0.032)		

† Sample size 1000 and 500 replicates. Shown are the mean (with standard deviations in parentheses) parameter estimates, empirical coverage probabilities of nominal 95% confidence intervals and mean-squared errors MSE.

In a second simulation study data were generated from a Weibull model and analysed by using four methods. To reduce the computation time a simpler model was chosen with a random intercept only and using a single binary covariate. The Weibull parameters were based on a Weibull fit to cystic fibrosis survival, with intercept −4.2 and shape 1.2. Other aspects of the model that was used to generate data were the same as in the previous simulation study, except times were censored at *t*=6 to enable us straightforwardly to carry out different discretizations of the timescale. Each simulated data set was analysed by using

1. a longitudinal model with a random intercept (so ignoring the survival data),
2. the R package joineR to fit a joint model with a shared random intercept and proportional hazards survival model,
3. the discrete time method that is described in this paper with six time intervals and
4. the discrete time method with three time intervals (note that our sequential probit model is misspecified for this scenario).

Table3 shows the results for the longitudinal model and the joint model fitted by using joineR. Fitting the longitudinal model separately gives slightly biased estimates for the longitudinal slope parameter and binary covariate. The joint model fit using joineR, however, gives parameter estimates with less bias and with good coverage, as would be expected because the data were generated under the same model as used in fitting. Table4 shows results by using the discrete time model with six and three time intervals. Here the model that was used to generate the survival data and the model used to fit the survival data are not equivalent, and so survival parameters estimated by the discrete time model cannot be compared with true values of the survival parameters. The coverage probabilities and mean-squared error could not therefore be calculated for these parameters. We can, however, see that the directions of the survival parameter estimates agree with those of the true model; positive parameter estimates in the discrete time model are in accordance with negative estimates in the true model because the former are linked to the probability of survival rather than the hazard of an event. Comparing longitudinal parameters, the standard errors are similar to the joineR results. Again, survival parameters are not directly comparable between the two models because the survival parameters of the six-interval model relate to the probability of surviving 1 year, and the parameters of the three-interval model to the probability of surviving 2 years. We would expect covariate effects to be similar, however, as is indeed the case. Comparing standard errors of survival parameter estimates, we find that the standard errors are slightly larger for the coarser time discretization.

**Table 3:** Simulation results from a Weibull model†

*Parameter*	*True*	*Results for longitudinal model*			*Results for joineR*		
	*value*						
		*Mean*	*Coverage*	*MSE*	*Mean*	*Coverage*	*MSE*
*Longitudinal*							
Intercept	90	90.213 (1.133)	0.948	1.326	89.982 (1.106)	0.954	1.222
Time	−1.7	−1.628 (0.061)	0.794	0.009	−1.698 (0.061)	0.954	0.004
Sex (males)	2	1.806 (1.578)	0.964	2.522	1.944 (1.581)	0.946	2.497
*Survival*							
Sex (males)	−0.3	—	—	—	−0.300 (0.196)	0.958	0.038
*γ*_1_	−0.1	—	—	—	−0.100 (0.005)	0.926	0.000
*Others*							
*ν*	7	7.007 (0.086)	0.928	0.007	6.999 (0.082)	0.946	0.007
*σ*_1_	25	24.789 (0.577)	0.824	0.377	24.977 (0.547)	0.956	0.300

† Sample size 1000 and 500 replicates. Shown are the mean (with standard deviations in parentheses) parameter estimates, empirical coverage probabilities of nominal 95% confidence intervals and mean-squared errors MSE. For the longitudinal model standard errors were calculated for variance parameters by using bootstrapping.

**Table 4:** Simulation results from a Weibull model†

*Parameter*	*True*	*Results for discrete time model,*			*Results for discrete time model,*		
	*value*	*6 intervals*			*3 intervals*		
		*Mean*	*Coverage*	*MSE*	*Mean*	*Coverage*	*MSE*
*Longitudinal*							
Intercept	90	90.034 (1.108)	0.958	1.225	90.041 (1.105)	0.960	1.221
Time	−1.7	−1.700 (0.061)	0.956	0.004	−1.697 (0.061)	0.954	0.004
Sex (males)	2	1.952 (1.584)	0.947	2.505	1.952 (1.581)	0.948	2.497
*Survival*							
Intercept	—	2.150 (0.122)	—	—	1.813 (0.132)	—	—
Time	—	−0.038 (0.024)	—	—	−0.039 (0.029)	—	—
Sex (males)	—	0.163 (0.110)	—	—	0.183 (0.126)	—	—
*γ*_1_	—	0.054 (0.004)	—	—	0.061 (0.004)	—	—
*Others*							
*ν*	7	6.998 (0.082)	0.945	0.007	7.000 (0.082)	0.948	0.007
*σ*_1_	25	24.932 (0.548)	0.958	0.303	24.908 (0.546)	0.958	0.305

† Sample size 1000 and 500 replicates. Shown are the mean (with standard deviations in parentheses) parameter estimates, empirical coverage probabilities of nominal 95% confidence intervals and mean-squared errors MSE.

## 5. Efficiency under coarsening at random

Although we allow the longitudinal measurements to be obtained in continuous or discrete time, we have required the event time data either originally to be measured on a discrete scale or to be placed on a discrete scale through coarsening at random via artificial interval censoring. We assume that our discrete time model is correct and so—because we use exact likelihood—our estimates are consistent and fully efficient given the data that we have chosen to use. However, discretization will affect the efficiency of an analysis, which is considered briefly in this section and expanded on in an on-line supplementary document. Here we concentrate only on survival analysis and omit much of the detail: this is provided together with further examples in the supplementary material. The supplementary material also includes a separate study into loss of information caused by discretization in the presence of random effects.

Let *T* be the continuous event time. Assume type 1 censoring at a maximum follow-up time *τ*. The follow-up interval (0,*τ*] is partitioned into *m* disjoint intervals, with boundaries 0=*t*_0_<*t*_1_<*t*_2_<…<*t*_*m*_=*τ*. Let *S* denote the interval within which *T* falls, with *S*=*m*+1 if *T* is censored at *τ*. Define *W*=(*T*−*t*_*s*−1_)/(*t*_*s*_−*t*_*s*−1_), which is the within-interval information on a (0,1) scale. Note that there is a one-to-one correspondence between *T* and (*S*,*W*). We shall investigate the loss of efficiency that is caused by ignoring *W*.

The sequential probit model (1) is assumed for event probabilities within each interval *j*, for *j*=1,2,…,*m*, with time constant covariates and covariate effects, but possibly time varying intercepts:

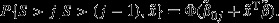
We assume for simplicity that the conditional within-interval distribution of event times is the same for all intervals. Let the corresponding probability density function be 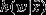, which will usually depend on 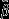 (and perhaps other parameters). Information on 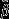 from the within-interval distribution of event times provides the extra efficiency for the complete-data analysis. To illustrate, assume that

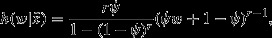
where 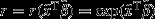 and 0<*ψ*<1. This is the within-interval distribution that arises if a Weibull distribution is discretized.

The information on 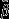 that is brought by *W* depends on how strongly 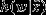 depends on 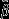. We can measure this through the curvature

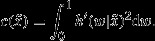
Given this set-up, the information that is associated with the marginal likelihood based only on *S* can be derived and compared with that from the full likelihood given both *S* and *W*. Table5 provides some numerical values for the ratio of asymptotic variances of maximum likelihood estimators. For this example we took a single binary covariate and set 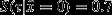 and 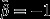. Since 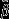 is fixed, changing *m* changes 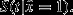 The final row in Table5 gives 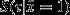 for each *m*. Intercepts 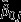 were chosen by assuming equal failure probabilities within each interval at 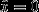.

**Table 5:** Efficiency of the coarsened-at-random analysis compared with the complete-data analysis†

*ψ*	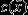	*Results for the following values of m:*			
		*m*=3	*m*=5	*m*=7	*m*=9
0.9	9.75	0.919	0.930	0.936	0.939
0.8	2.28	0.958	0.964	0.967	0.969
0.7	0.91	0.976	0.979	0.981	0.982
0.6	0.44	0.986	0.988	0.989	0.990
0.5	0.22	0.992	0.993	0.994	0.994
0.4	0.11	0.996	0.996	0.997	0.997
0.3	0.05	0.998	0.998	0.998	0.998
0.2	0.02	0.999	0.999	0.999	0.999
0.1	0.00	1.000	1.000	1.000	1.000
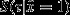		0.201	0.152	0.123	0.104

† Values in the main block are the ratios of asymptotic variance estimators for 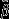 without and with *W*.

Some calibration based on curvature of discretized Weibull distributions is provided in the on-line supplementary material. The curvature in Table5 at *ψ*>0.7 is higher than anything seen for the Weibull discretizations that we considered. Even so, the efficiency was more than 90% in all simulation scenarios and was more than 97% in more realistic cases. We obtained similar results for other 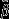, and when we fixed 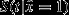 and allowed 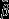 to vary with *m*.

## 6. Application: disease progression and survival in cystic fibrosis patients

We now apply our methods to data on repeated measurements of lung function in cystic fibrosis patients, some of whom died during follow-up. The data were from the UK Cystic Fibrosis registry and cover the years 1999–2010. Cystic fibrosis is the commonest serious inherited disease among Caucasian populations, and most patients die as a result of progressive respiratory disease (Davies and Alton, 2009). Previous examples of longitudinal modelling applied to cystic fibrosis data include Schluchter *et al*. (2002), van Diemen *et al*. (2011) and Taylor-Robinson *et al*. (2012). Here, we fit a range of joint models to data from the 1980–1984 birth cohort of the UK registry, conditional on survival to capture in the registry in 1999. The data set included 1231 patients who were alive in 1999, of whom 230 died during the course of follow-up to 2010. Our repeated measurement outcome is per cent predicted forced expiratory volume in 1 s, %FEV1, which is used as a measure of lung function and is recognized as a key outcome measure in cystic fibrosis (Rosenfeld *et al*., 2005; Davies and Alton, 2009; Orens, 2006). Measurements were taken approximately annually, with some variation between patients. The number of measurements per person varied from 1 to 17. The covariates used were sex and age at initial visit. The timescale was the number of years since the initial visit, with initial age fitted as a separate covariate to allow for left truncation and cohort effects. For the survival model the timescale was discretized into yearly intervals. At the initial visit 54.2% of patients were male, the mean age was 18.9 years (standard deviation SD 2.8) and the mean %FEV1 was 67.1 (SD 25.3).

Table6 shows the results from fitting four random-effects models to the data: a stationary Gaussian process SGP, a stationary Gaussian process with one time lag in the survival model, lagged SGP, a random intercept and slope model IS and a stationary Gaussian process plus random intercept and slope, SGP+IS. The positive effect of the sex covariate in the survival model indicates that males have a significantly better probability of survival than females. The estimated covariate effects are in agreement with expectations for cystic fibrosis patients, with older patients tending to have poorer lung function and survival than younger patients and males tending to have better lung function and to survive longer than females.

**Table 6:** Joint model fits of the data from cystic fibrosis patients†

*Parameter*	*Results for the following models:*			
	*SGP*	*Lagged SGP*	*IS*	*SGP + IS*
*Longitudinal*				
Intercept	74.899 (4.868)	74.904 (4.948)	76.683 (5.069)	76.669 (4.406)
Time	−1.502 (0.074)	−1.502 (0.074)	−1.762 (0.082)	−1.652 (0.080)
Age at *t*_0_	−0.454 (0.250)	−0.454 (0.256)	−0.577 (0.262)	−0.568 (0.218)
Sex (males)	0.786 (1.415)	0.785 (1.377)	1.759 (1.420)	1.506 (1.143)
*Survival*				
Intercept	2.964 (0.344)	2.962 (0.355)	3.290 (0.422)	3.441 (0.276)
Time	−0.023 (0.011)	−0.023 (0.012)	−0.050 (0.014)	−0.052 (0.013)
Age at *t*_0_	−0.021 (0.017)	−0.021 (0.018)	−0.034 (0.020)	−0.034 (0.013)
Sex (males)	0.234 (0.084)	0.234 (0.084)	0.268 (0.091)	0.287 (0.097)
*γ*	0.037 (0.002)	0.037 (0.003)	—	0.040 (0.009)
*γ*_lag_	—	0.000 (0.001)	—	—
*γ*_1_	—	—	0.035 (0.002)	0.036 (0.004)
*γ*_2_	—	—	0.270 (0.032)	0.418 (0.084)
*Others*				
*ν*	7.235 (0.100)	7.235 (0.100)	8.806 (0.084)	7.231 (0.108)
*σ*_*u*_	25.081 (0.485)	25.081 (0.485)	—	12.832 (1.655)
*σ*_1_	—	—	24.240 (0.523)	20.486 (1.211)
*σ*_2_	—	—	2.152 (0.074)	1.249 (0.108)
*ρ*_SGP_	0.969 (0.002)	0.969 (0.002)	—	0.890 (0.031)
*ρ*_IS_	—	—	−0.066 (0.042)	0.218 (0.086)
AIC	49551.96	49553.96	49878.47	49522.25

† The random-effects models fitted were a stationary Gaussian process SGP, a stationary Gaussian process with one time lag in the survival model, lagged SGP, a random intercept and slope model IS and a stationary Gaussian process plus random intercept and slope, SGP+IS. For each model estimated parameter values are presented with standard errors in parentheses, and the Akaike information criterion AIC.

For the SGP model, the positive estimate of *γ* means that better lung function is associated with improved survival in cystic fibrosis patients. For the lagged SGP model there is no evidence that lung function during the previous time interval affects survival, after adjusting for current lung function. The estimated parameters *γ*_1_ and *γ*_2_ from model IS indicate that patients with higher intercepts and less negative slopes of %FEV1 are more likely to survive. Comparing the fit to the data of all four models by using the Akaike information criterion AIC suggests that the model providing the best fit is the model combining both a stationary Gaussian process and a random intercept and slope.

One way to facilitate interpretation of the parameters of the probit model is suggested in Table7. Here we explore the effect of changing a parameter value on the probability of death in a time interval, while all other parameters are fixed to their default (i.e. mean or baseline) values. For example, the probability of death in year 1 for a woman with default covariate values is 0.003, compared with a probability of 0.001 for a man with the same characteristics.

**Table 7:** Interpretation of probit parameters†

*Survival parameter*	*Default*	*Test*	*Probability*
	*value*	*value*	*of death*
*Time effects*			
Year 1			0.003
Year 2			0.003
Year 3			0.004
Year 4			0.004
Year 5			0.005
Year 6			0.006
Year 7			0.006
Year 8			0.007
Year 9			0.009
Year 10			0.010
*Covariate effects*			
Age at *t*_0_ (years)	18.9	23.9	0.004
Sex	Female	Male	0.001
Stationary Gaussian process *U*	0	−10	0.008
Random intercept *U*_1_	0	−10	0.007
Random slope *U*_2_	0	−2	0.025

† Default values are the mean or baseline value of each parameter. For the effect of time, the probabilities of death in each time interval are given, at default values of other parameters. For other covariate effects the probability of death in the first year is given, when the parameter indicated is changed to the test value, and all other parameters take their default values.

## 7. Discussion

We have described a method that combines flexibility of model specification with tractability of likelihood. It can be applied to repeated measurement data with dropout occurring between scheduled measurement times, or to the joint analysis of longitudinal and survival time data, provided that the survival timescale is discrete, or can realistically be discretized. It avoids the need for numerical approximation of an integral over the random effects or EM methods.

The method allows the fitting of models with more complex random effects because the number of random-effects terms in the survival model is not constrained by computational time. This may come at the cost of discretizing the timescale, but simulation studies and analysis of special cases suggest that, although some information is inevitably lost, parameter estimation may not be greatly influenced by discretization. In the on-line supplementary material we show that there is no loss of information if the survival functions are linear between discrete time points. Hence a discretization that keeps approximate linearity is recommended. Our evidence shows that there can be little loss of efficiency even in the presence of quite strong non-linearity. In practice there may often be a natural discrete timescale. For example the cystic fibrosis patients had visits around once a year, and an annual discretization seems suitable because shorter-term fluctuations in the underlying continuous time hazard will be poorly identified.

Computational time is driven primarily by the need to calculate multivariate normal probabilities, which can be time consuming for high dimensional data. But the intercept and slope model fitted in around a third of the time required by the R package joineR using 200 bootstraps to calculate standard errors. Alternative approaches to maximizing the likelihood may enable computation time to be further reduced, e.g. by iterating between a Newton–Raphson step for covariate parameters and numerical maximization over variance parameters. Alternatively a prespecified number of steps of an EM algorithm could be used to obtain initial values, as in the R package JM (Rizopoulos, 2010). Even with our current procedure we could fit fairly high dimensional random-effects models in simulations and the cystic fibrosis application (i.e. model SGP, which has a random effect associated with each time interval), whereas current software (R packages JM and joineR) is limited to simple random-effects models or relies on rough approximations of multiple integrals over random effects. Inclusion of stationary Gaussian process random effects led to a marked improvement in AIC for the cystic fibrosis data (Table6).

Without readily available software it is unlikely that any reasonably sophisticated statistical methodology will find wide use in applications. Our intention is to develop R software to implement the technique and so to supplement the joint models that can be fitted in JM and joineR. We have provided R code to calculate the likelihood as supplementary material available from http://wileyonlinelibrary.com/journal/rss-datasets

## Appendix A: Evaluation of the likelihood

### A.1. Evaluation of *L*_1_

The longitudinal and random-effects components of the likelihood together are

This can be rearranged as

where

In turn

10

where *ϕ*^(*p*)^(·;*h*,*H*^−1^) denotes the *p*-dimensional Gaussian density with mean vector *h* and variance matrix *H*^−1^. Collecting all unknown parameters into vector *θ*, equation 10 can be written as

11

where

### A.2. Evaluation of *L*_2_

The full likelihood, integrating over random effects, is

12

Consider the integral in likelihood (12) at *δ*=0, i.e.

13

To perform the integration we use the skew normal result (4), which is

14

We can convert the right-hand side of equation 14 to the form (13) by sequentially setting

then

and finally . Hence

The contribution of the integral *L*_2_(*θ*;*y*,*s*,*δ*=1) in expression (12) at *δ*=1 can be obtained similarly, to give

## Appendix B: Regularity conditions

Collect all unknown parameters into a vector *θ*, with true value *θ*_0_ in the interior of a compact set Ω. Let the observed data on individual *i* be *z*_*i*_, consisting of longitudinal responses, event times, censoring indicator and covariates. Write  for the likelihood contribution of subject *i*, for *i*=1,2,…,*n*.

From Cramér's theorem (Ferguson, 1996; Kotz and Johnson, 1985) the following conditions are sufficient for the maximum likelihood estimator *θ* to converge in distribution to a Gaussian variable, centred at *θ*_0_ and with variance given by the inverse expected information.

### Condition 1

*Z*_1_,*Z*_2_,…,*Z*_*n*_ are independent and identically distributed.

### Condition 2

There is an open subset *ω* of *Θ* such that for all *θ* ∈ *ω* and almost all *z* the second partial derivatives  exist and may be passed under the integral sign in

where *ν*(*z*) is the measure associated with *Z*.

### Condition 3

The Fisher information

is positive definite for all *θ* in *ω*.

### Condition 4

There are functions *K*(*z*), independent of *θ*, such that each component of  is bounded by *K*(*z*) uniformly for *θ* ∈ *ω*.

### Condition 5

*f*(*z*|*θ*) is identifiable, i.e. if  almost everywhere then *θ*_1_≠*θ*_2_.

### Condition 6

The support of *f*(*z*|*θ*) does not change with *θ*.

Conditions 1-4 are standard given our fully parametric Gaussian random-effects model, provided that we assume (as we do) that the longitudinal measurement schedule {*t*_*j*_}, the number of discrete intervals *m* and the selection of covariates are all fixed and ancillary. Further, all variance parameters are finite and strictly positive.

Conditions 5 and 6 need more attention in the presence of missing data. We need to ensure that as the sample size increases there are sufficient observations to identify the survival probability for each discrete time interval, and all parameters in the longitudinal model. Hence we make the following additional assumptions.

### Assumption 1

Let *θ*_*Y*_ be the subset of *θ* that appears in the marginal distribution  of *Y* implied by expressions (2) and (3). If *γ*=0, condition 5 holds for  and *θ*_*Y*_.

### Assumption 2

Any right censoring of event times is independent (Andersen *et al*. (1993), definition II.2.1).

### Assumption 3

There exists *c*>0 such that *P*(Δ=0)<1−*c*, where Δ is the censoring indicator.

### Assumption 4

There exist *c*_1_>0 and *c*_2_>0, independent of *θ*, such that

for each  and all  in the appropriate subset of *ω*.

### Assumption 5

For all  and all *U*, the second-partial-derivative matrix

is of full rank, where *θ*_*S*_ ∈ *ω* denotes the combined parameters  appearing in the event time model (7).

Recalling that the parameter vector *γ* links the longitudinal and event processes, assumption 1 ensures that the longitudinal model is well defined. The independent censoring assumption referred to in assumption 2 is essentially a requirement that there is no prognostic information in knowing that an event time is censored. Assumption 3 ensures that all event times in  are observable and assumption 4 makes sure that in large samples there are both events and survivors for each interval. Finally assumption 5 ensures identifiability of  and *γ*.

In assumptions 4 and 5 we have assumed a structural model in which the covariates are considered to be independent and identically distributed random variables. In the functional case we need alternatives of the form

and

is of full rank, in each case for *n*>*n*_0_ say.

## Supporting Information

‘Supplementary material 1: efficiency under coarsening at random’.
