# Supplementary material for: Joint modelling of repeated measurements and time-to-event outcomes: flexible model specification and exact likelihood inference
Source: J R Stat Soc Series B Stat Methodol. 2014 Apr 8;77(1):131–48. doi: 10.1111/rssb.12060 (PMC4384944; doi:10.1111/rssb.12060)
Supplement: Supplementary file 1 — ‘Supplementary material 1: efficiency under coarsening at random’. [file rssb0077-0131-sd1.pdf]

Joint Modelling of Repeated Measurements and Time-to-Event Outcomes: Flexible Model Specification and Exact Likelihood Inference
Supplementary Material 1: Efficiency under Coarsening at Random

Jessica Barrett, Peter Diggle, Robin Henderson and David Taylor-Robinson

September 20, 2013

We assume our discrete-time model is correct and so - because we use exact likelihood - our estimates are consistent and fully efficient given the data we have chosen to use, ie survival times coarsened at random through artificial interval censoring. Hence at issue is loss of efficiency only. A full efficiency analysis will be model (or model class) specific and almost certainly intractable for joint models with random effects. However, we can make useful progress if we consider a) a survival analysis only, or b) the additional information that within-interval information provides on the random effects that link the longitudinal and survival parts.

1 Survival Analysis

Let T be the continuous event time. Assume Type 1 censoring at a maximum follow-up time τ. Extension to additional random censoring is feasible but not central to discussion of efficiency. The follow-up interval $(0, \tau]$ is partitioned into m disjoint intervals, with boundaries $0 = t_0 < t_1 < t_2 < \ldots < t_m = \tau$. Let S denote the interval within which T falls, with $S = m+1$ if T is censored at τ. Define $W = (T - t_{s-1})/(t_s - t_{s-1})$, which is the within-interval information on a (0,1) scale. Note that there is a one-to-one correspondence between T and (S, W). We will investigate the loss of efficiency caused by ignoring W.

The sequential probit model in (1) of the main paper is assumed for event probabilities within each interval j, for $j = 1, 2, \ldots, m$, with time-constant covariates and covariate effects, but possibly time-varying intercepts:

$$P(S > j | S > (j-1), \tilde{x}) = \Phi\left(\tilde{\beta}_{0j} + \tilde{x}^T \tilde{\beta}\right).$$

In the numerical work later, we take scalar binary $\tilde{x}$, fix $P(S = (m+1)|\tilde{x} = 0)$ and assume equal failure probabilities within each interval at $\tilde{x} = 0$. We then either fix $\tilde{\beta}$ or fix $P(S = (m+1)|\tilde{x} = 1)$ and again assume equal failure probabilities for each interval at $\tilde{x} = 1$. As m varies we cannot have both of these options: with fixed $\tilde{\beta}$ then $P(S = (m+1)|\tilde{x})$ changes with m; with fixed $P(S = (m+1)|\tilde{x})$ then $\tilde{\beta}$ must change with m in our set-up.

We assume for simplicity that the conditional within-interval distribution of event times is the same for all intervals. Let the corresponding probability density function be $h(w|\tilde{x})$, which will usually depend on $\tilde{\beta}$ (and perhaps other parameters). Information on $\tilde{\beta}$ from the within-interval distribution of event times provides the extra efficiency for the complete data (not coarsened) analysis.

1.1 Likelihood analysis with coarsening

Assume a generic subject either has an event in interval s, for $s = 1, 2, \ldots, m$, or is censored at τ, in which case we set $s = m + 1$. Let δ be an indicator of event ($\delta = 1$) or censoring ($\delta = 0$).

The likelihood contribution is

$$L_C = \left(\prod_{j=1}^{s-1} \Phi\left(\tilde{\beta}_{0j} + \tilde{x}^T \tilde{\beta}\right) \right) \left(1 - \Phi\left(\tilde{\beta}_{0s} + \tilde{x}^T \tilde{\beta}\right)\right)^{\delta}.$$

The log-likelihood is

$$\ell_C = \left(\sum_{j=1}^{s-1} \log(\Phi\left(\tilde{\beta}_{0j} + \tilde{x}^T \tilde{\beta}\right)) \right) + \delta \log\left(1 - \Phi\left(\tilde{\beta}_{0s} + \tilde{x}^T \tilde{\beta}\right)\right).$$

For $j = 1, 2, \ldots, m$:

$$\frac{\partial \ell_C}{\partial \tilde{\beta}_{0j}} = I(s > j) \frac{\phi\left(\tilde{\beta}_{0j} + \tilde{x}^T \tilde{\beta}\right)}{\Phi\left(\tilde{\beta}_{0j} + \tilde{x}^T \tilde{\beta}\right)} - I(s = j) \frac{\phi\left(\tilde{\beta}_{0j} + \tilde{x}^T \tilde{\beta}\right)}{1 - \Phi\left(\tilde{\beta}_{0j} + \tilde{x}^T \tilde{\beta}\right)},$$

remembering that we have assumed no censoring before τ so that if $s < m + 1$ then an event must have occurred. Also

$$\frac{\partial \ell_C}{\partial \tilde{\beta}} = \left[\left(\sum_{j=1}^{s-1} \frac{\phi\left(\tilde{\beta}_{0j} + \tilde{x}^T \tilde{\beta}\right)}{\Phi\left(\tilde{\beta}_{0j} + \tilde{x}^T \tilde{\beta}\right)} \right) - \delta \frac{\phi\left(\tilde{\beta}_{0s} + \tilde{x}^T \tilde{\beta}\right)}{1 - \Phi\left(\tilde{\beta}_{0s} + \tilde{x}^T \tilde{\beta}\right)} \right] \times \tilde{x},$$

$$\frac{\partial^2 \ell_C}{\partial \tilde{\beta}_{0j}^2} = -I(s>j)\frac{\phi\left(\tilde{\beta}_{0j}+\tilde{x}^T\tilde{\beta}\right)}{\Phi\left(\tilde{\beta}_{0j}+\tilde{x}^T\tilde{\beta}\right)}\left(\tilde{\beta}_{0j}+\tilde{x}^T\tilde{\beta}+\frac{\phi\left(\tilde{\beta}_{0j}+\tilde{x}^T\tilde{\beta}\right)}{\Phi\left(\tilde{\beta}_{0j}+\tilde{x}^T\tilde{\beta}\right)}\right)$$
$$+I(s=j)\frac{\phi\left(\tilde{\beta}_{0j}+\tilde{x}^T\tilde{\beta}\right)}{1-\Phi\left(\tilde{\beta}_{0j}+\tilde{x}^T\tilde{\beta}\right)}\left(\tilde{\beta}_{0j}+\tilde{x}^T\tilde{\beta}-\frac{\phi\left(\tilde{\beta}_{0j}+\tilde{x}^T\tilde{\beta}\right)}{1-\Phi\left(\tilde{\beta}_{0j}+\tilde{x}^T\tilde{\beta}\right)}\right),$$

$$\frac{\partial^2 \ell_C}{\partial \tilde{\beta}_{0j}\partial \tilde{\beta}_{0k}} = 0 \quad j \neq k,$$

$$\frac{\partial^2 \ell_C}{\partial \tilde{\beta}\partial \tilde{\beta}^T} = \left[-\sum_{j=1}^{s-1}\frac{\phi\left(\tilde{\beta}_{0j}+\tilde{x}^T\tilde{\beta}\right)}{\Phi\left(\tilde{\beta}_{0j}+\tilde{x}^T\tilde{\beta}\right)}\left(\tilde{\beta}_{0j}+\tilde{x}^T\tilde{\beta}+\frac{\phi\left(\tilde{\beta}_{0j}+\tilde{x}^T\tilde{\beta}\right)}{\Phi\left(\tilde{\beta}_{0j}+\tilde{x}^T\tilde{\beta}\right)}\right)\right.$$
$$\left.+\delta\frac{\phi\left(\tilde{\beta}_{0j}+\tilde{x}^T\tilde{\beta}\right)}{1-\Phi\left(\tilde{\beta}_{0j}+\tilde{x}^T\tilde{\beta}\right)}\left(\tilde{\beta}_{0j}+\tilde{x}^T\tilde{\beta}-\frac{\phi\left(\tilde{\beta}_{0j}+\tilde{x}^T\tilde{\beta}\right)}{1-\Phi\left(\tilde{\beta}_{0j}+\tilde{x}^T\tilde{\beta}\right)}\right)\right]\times\tilde{x}\tilde{x}^T.$$

And finally

$$\frac{\partial^2 \ell_C}{\partial \tilde{\beta}_{0j}\partial \tilde{\beta}} = \left[-I(s>j)\frac{\phi\left(\tilde{\beta}_{0j}+\tilde{x}^T\tilde{\beta}\right)}{\Phi\left(\tilde{\beta}_{0j}+\tilde{x}^T\tilde{\beta}\right)}\left(\tilde{\beta}_{0j}+\tilde{x}^T\tilde{\beta}+\frac{\phi\left(\tilde{\beta}_{0j}+\tilde{x}^T\tilde{\beta}\right)}{\Phi\left(\tilde{\beta}_{0j}+\tilde{x}^T\tilde{\beta}\right)}\right)\right.$$
$$\left.+I(s=j)\frac{\phi\left(\tilde{\beta}_{0j}+\tilde{x}^T\tilde{\beta}\right)}{1-\Phi\left(\tilde{\beta}_{0j}+\tilde{x}^T\tilde{\beta}\right)}\left(\tilde{\beta}_{0j}+\tilde{x}^T\tilde{\beta}-\frac{\phi\left(\tilde{\beta}_{0j}+\tilde{x}^T\tilde{\beta}\right)}{1-\Phi\left(\tilde{\beta}_{0j}+\tilde{x}^T\tilde{\beta}\right)}\right)\right]\times\tilde{x}.$$

We can use the second derivatives together with the known distribution of discrete event times to obtain the asymptotic expected information (and variance matrix) under coarsening at random.

1.2 Distribution of W

First we note that a simple approach would be to interpolate:

$$P(T>t|\tilde{x}) = \left(\prod_{j=1}^{k-1}\Phi\left(\tilde{\beta}_{0j}+\tilde{x}^T\tilde{\beta}\right)\right)\left(\frac{t_k-t}{t_k-t_{k-1}}+\frac{t-t_{k-1}}{t_k-t_{k-1}}\Phi\left(\tilde{\beta}_{0k}+\tilde{x}^T\tilde{\beta}\right)\right) \quad \text{for } t_{k-1}<t\leq t_k.$$

The associated density is:

$$f(t|\tilde{x}) = \left(\prod_{j=1}^{k-1} \Phi\left(\tilde{\beta}_{0j} + \tilde{x}^T \tilde{\beta}\right) \right) \frac{1 - \Phi\left(\tilde{\beta}_{0k} + \tilde{x}^T \tilde{\beta}\right)}{t_k - t_{k-1}},$$

again for $t_{k-1} < t \leq t_k$. Hence conditional on failing in interval k

$$f(t|\tilde{x}, S = k) = \frac{f(t|\tilde{x})}{P(S > t_{k-1}|\tilde{x}) - P(S > t_k|\tilde{x})} = \frac{1}{t_k - t_{k-1}},$$

and there is no information in the within-interval value of the event time T. In this case our coarsened at random analysis is fully efficient. Thus if we discretise so that the survival distributions are (approximately) linear then our discrete-time analysis is (almost) efficient.

To investigate the effect of coarsening there must be an effect of $\tilde{x}^T \tilde{\beta}$ on the distribution of the within-interval value W. As stated, we will assume that W has the same distribution within all discrete intervals, with density $h(w|\tilde{x})$. Note that we can always transform so that $h(w|\tilde{x} = 0)$ is the U(0,1) density.

We now need to introduce non-uniform distributions for other $\tilde{x}$. We assume

$$h(w|\tilde{x}) = \frac{r\psi}{(1 - (1 - \psi)^r)} \left(\psi w + 1 - \psi\right)^{r-1},$$

where $r = r(\tilde{x}^T \tilde{\beta}) = \exp(\tilde{x}^T \tilde{\beta})$ and $0 < \psi < 1$. This is the within-interval distribution that arises if a Weibull distribution is discretised (Appendix). Note that high $\tilde{x}^T \tilde{\beta}$ is associated with high discrete-time survival and we have kept the same ordering within intervals: if $r > 1$ then high values of W are more likely, the opposite for $r < 1$.

As required the density is uniform at $\tilde{x}^T \tilde{\beta} = 0$, and also at $\psi = 0$. We will use curvature

$$c(\tilde{x}) = \int_0^1 \left(h'(w|\tilde{x})\right)^2 dw$$

as a measure of non-uniformity. For the chosen $h(w|\tilde{x})$ we can show that

$$c(\tilde{x}) = \begin{cases} \frac{r^2(r-1)^2\psi^3}{2r-3} \left[\frac{1-(1-\psi)^{2r-3}}{(1-(1-\psi)^r)^2}\right] & r \neq 3/2, \\ -\frac{9}{16} \frac{\psi^3}{(1-(1-\psi)^{3/2})^2} \log(1-\psi) & r = 3/2. \end{cases}$$

Some calibration is provided by specimen values of $c(\tilde{x})$ given in the Appendix for discretised Weibull distributions. Values around 2 are quite extreme.

1.3 Likelihood analysis without coarsening

Since we only allow censoring at the final point τ, the additional log-likelihood, score and information contributions associated with W are

$$\ell_W = I(s \leq m)\left[\log(r) + \log(\psi) - \log\left(1 - (1-\psi)^r\right) + (r-1)\log\left(w\psi + 1 - \psi\right)\right],$$

$$\frac{\partial \ell_W}{\partial \tilde{\beta}} = -I(s \leq m)\left[\frac{1}{r} + \frac{(1-\psi)^r \log(1-\psi)}{(1-(1-\psi)^r)} + \log\left(w\psi + 1 - \psi\right)\right] \times \tilde{x}r,$$

and

$$\begin{aligned}\frac{\partial^2 \ell_W}{\partial \tilde{\beta} \partial \tilde{\beta}^T} &= I(s \leq m)\left[\frac{(1-\psi)^r \log(1-\psi)}{(1-(1-\psi)^r)} + \log\left(w\psi + 1 - \psi\right)\right. \\ &\quad \left. \frac{r(1-\psi)^r(\log(1-\psi))^2}{(1-(1-\psi)^r)} + \frac{r\left((1-\psi)^r \log(1-\psi)\right)^2}{\left(1-(1-\psi)^r\right)^2}\right] \times \tilde{x}\tilde{x}^T r.\end{aligned}$$

For the expected information we need

$$E[\log\left(W\psi + 1 - \psi\right)] = \frac{1}{r(1-(1-\psi)^r)}\left[(1-\psi)^r\left\{1 - r\log(1-\psi)\right\} - 1\right]$$

1.4 Example

Table 1 provides examples of the efficiency of the coarsened at random analysis compared with the complete-data analysis. For this example we took a single binary covariate and set $S(\tau|\tilde{x}=0) = 0.7$ and $\tilde{\beta} = -1$. Since $\tilde{\beta}$ is fixed, changing m changes $S(t|\tilde{x}=1)$. The final row in the table gives $S(\tau|\tilde{x}=1)$ for each m.

Survival curves at $\psi = 0.9$ are shown in Figure 1. Recall that the distribution of W has to differ between $\tilde{x}=0$ and $\tilde{x}=1$ if the coarsening is to lose any information. For calibration, it is useful to look at the Weibull results in the Appendix. The curvature at $\psi = 0.9$ is higher than anything seen for the Weibull discretisations. And we assume that applies within *all* intervals and so Figure 1 corresponds to a rather extreme example. Even so, there is little loss of efficiency when we ignore the within-interval information. In all simulation scenarios the efficiency was more than 90%, and was more than 97% in more realistic cases. We obtained similar results for other $\tilde{\beta}$, and when we fixed $S(t|\tilde{x}=1)$ and allowed $\tilde{\beta}$ to vary with m.

ψ	$c(\tilde{x})$	$m=3$	$m=5$	$m=7$	$m=9$
0.9	9.75	0.919	0.930	0.936	0.939
0.8	2.28	0.958	0.964	0.967	0.969
0.7	0.91	0.976	0.979	0.981	0.982
0.6	0.44	0.986	0.988	0.989	0.990
0.5	0.22	0.992	0.993	0.994	0.994
0.4	0.11	0.996	0.996	0.997	0.997
0.3	0.05	0.998	0.998	0.998	0.998
0.2	0.02	0.999	0.999	0.999	0.999
0.1	0.00	1.000	1.000	1.000	1.000
	$S(\tau\|\tilde{x}=1)$	0.201	0.152	0.123	0.104

Table 1: Efficiency of coarsened at random analysis compared with complete-data analysis. Values in the main block are the ratios of asymptotic variance estimators for $\tilde{\beta}$ without and with W.

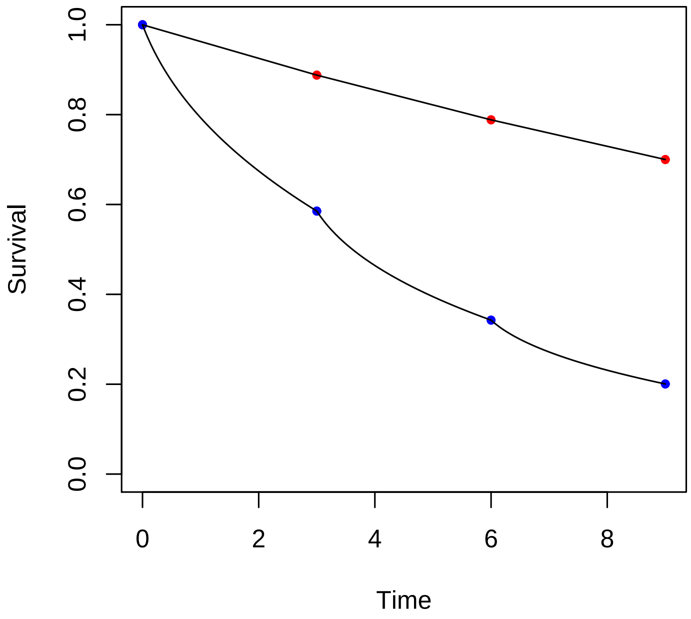

Figure 1: Example survival curves at $S(\tau|\tilde{x}=0)=0.7$, $\tilde{\beta}=-1$ (so $S(\tau|\tilde{x}=1)\simeq 0.2$) and $\psi=0.9$. Circles are fixed by the discrete time model, and the lines between by the within-interval model. The upper curve has $\tilde{x}=0$ and the lower has $\tilde{x}=1$.

2 Random Effects

We now turn to joint modelling and introduce a random effect U. The key comparison is between $f(U|S,W)$ and $f(U|S)$, where S is the discretised survival time.

We will consider a generic subject with scalar U. Because we are not now considering estimation, without loss we exclude covariates (or equivalently assume covariate effects are incorporated in other parameters). We will adapt the model considered so far, with the following additional assumptions.

1. $U \sim \mathrm{N}(0, \sigma^2)$.
2. Conditional on U, S has our sequential probit discrete distribution
$$f_{S|U}(s|u) = \left(\prod_{j=1}^{s-1} \Phi\left(\tilde{\beta}_{0j} + u\right) \right) \left(1 - \Phi\left(\tilde{\beta}_{0s} + u\right)\right)^{\delta}.$$
Note that high U is associated with high S.
3. Also conditional on U
$$f_{W|U}(w|u) = \frac{r\psi}{(1-(1-\psi)^r)} \left(\psi w + 1 - \psi\right)^{r-1}$$
where $r = \exp(u)$, so high U is associated with high W. Note that $(W|U=0) \sim U(0,1)$.
4. S and W are conditionally independent given U.

To illustrate, we again take m equally probable intervals to a maximum survival time of τ. We take $S(\tau|U=0) = 0.5$. To calibrate σ^2, consider $m = 3$.

σ	$S(\tau\|U=-\sigma)$	$S(\tau\|U=-\sigma)$
0.25	0.37	0.63
0.50	0.24	0.74
0.75	0.15	0.84
1.00	0.08	0.90

Since $\Phi(-1) = 1 - \Phi(1) \simeq 0.16$, the above shows that at eg $\sigma = 0.5$ about 16% of people will have survival probabilities to τ of 0.24 or less, and 16% will have survival probabilities of 0.74 or greater. For large σ the distribution of $S(\tau|U)$ becomes ever more concentrated at the boundaries. Choosing $\sigma = 0.5$ gives a strong but not unrealistically extreme distribution. Hence we set $\sigma = 0.5$ in the following.

Figure 2 illustrates for $m = 3$, $S(\tau|U=0) = 0.5$, $\psi = 0.9$ and $\sigma = 0.5$. Each subplot shows the marginal of U, the conditional given S, and the conditionals also given either low W or high W. The different subplots correspond to the four possible values of S. As expected, the

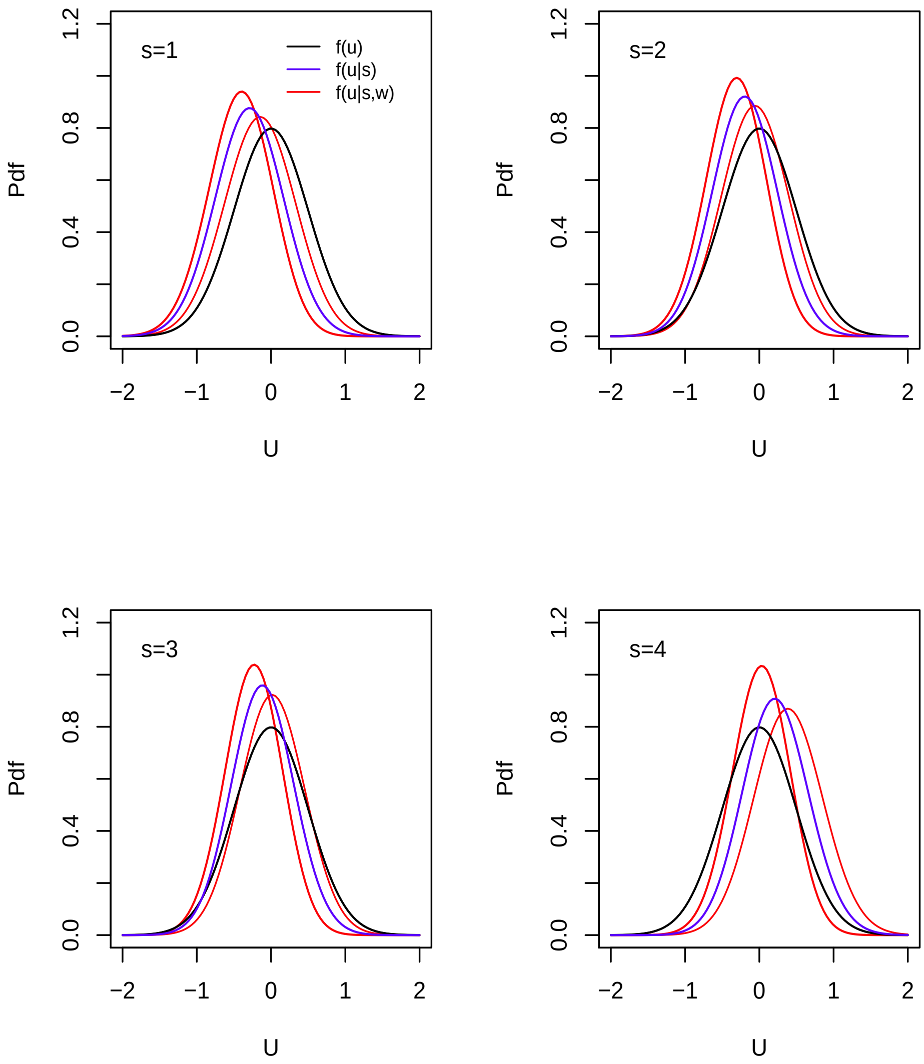

Figure 2: Distribution of random effect U: marginal, $f_U(u)$ (black line); given $S = s$, $f_{U|S}(u|S = s)$ (blue); and given $S = s$ and $W = 0.1$ or $W = 0.9$, $f_{U|S,W}(u|S = s, W = 0.1)$ (left red line) and $f_{U|S,W}(u|S = s, W = 0.9)$ (right red line). Parameters $m = 3$, $S(\tau|U = 0) = 0.5$, $\psi = 0.9$ and $\sigma = 0.5$.

$E[\text{Var}(U\|S)]/\text{Var}(U)$			
$m=3$	$m=5$	$m=7$	$m=9$
0.82	0.78	0.76	0.75

$E[\text{Var}(U\|S,W)]/E[\text{Var}(U\|S)]$				
ψ	$m=3$	$m=5$	$m=7$	$m=9$
0.9	0.94	0.94	0.94	0.94
0.8	0.96	0.96	0.96	0.96
0.7	0.97	0.97	0.97	0.98
0.6	0.98	0.98	0.98	0.98
0.5	0.99	0.99	0.99	0.99
0.4	0.99	1.00	0.99	0.99
0.3	1.00	1.00	1.00	1.00
0.2	1.00	1.00	1.00	1.00
0.1	1.00	1.00	1.00	1.00

Table 2: Ratios of posterior variances

distribution shifts to the left on conditioning on low S, and to the left (right) on additional conditioning on low (high) W. The changes are all rather modest however, even at this quite extreme value of ψ. Similar plots (not shown) were obtained for other values of m.

Table 2 compares posterior variances of the random effect given either S or (S, W). First we draw U from the marginal distribution, then we draw S and W from the appropriate conditionals given U. We calculate the variances of the distributions $f_{U|S}(u|S)$ and $f_{U|S,W}(u|S,W)$ and then average over the original U. Results are based on 5000 Monte Carlo simulations of U, S and W, with numerical integration for the posterior expectations. There is up to a 25 % reduction in variance once S is available, but further gain from W is less than 6%.

Finally Table 3 illustrates mean square prediction error (MSPE). The procedure is similar to that just used, but instead of variances we take mean square error between the posterior means of $f_{U|S}(u|S)$ or $f_{U|S,W}(u|S,W)$ and the value of U used to generate S and W, again with averaging over U. In the absence of conditioning the MSPE is just $\text{Var}(U) = \sigma^2$. The tables again look at ratios. Conclusions are unchanged: there is very little additional information in the within-interval value W, with reductions of only 6% even in some quite extreme situations.

$E[(E[U|S]-U)^2]/\mathrm{Var}(U)$			
$m=3$	$m=5$	$m=7$	$m=9$
0.41	0.39	0.38	0.38

$E[(E[U\|S,W]-U)^2]/E[(E[U\|S]-U)^2]$				
ψ	$m=3$	$m=5$	$m=7$	$m=9$
0.9	0.94	0.94	0.94	0.94
0.8	0.96	0.96	0.97	0.96
0.7	0.97	0.97	0.97	0.97
0.6	0.99	0.99	0.99	0.98
0.5	0.99	0.99	0.99	0.99
0.4	0.99	0.99	1.00	0.99
0.3	1.00	1.00	1.00	1.00
0.2	1.00	1.00	1.00	1.00
0.1	1.00	1.00	1.00	1.00

Table 3: Ratios of mean square prediction errors

Appendix: Partitioning a Weibull distribution

Consider Weibull survival T with a binary covariate and shape parameter ζ. We will choose rates by fixing $p_0 = S(\tau|\tilde{x}=0)$ and $p_1 = S(\tau|\tilde{x}=1)$. Hence we choose θ_0 and θ_1 such that

$$e^{-\theta_0 \tau^\zeta} = p_0, \qquad e^{-\theta_1 \tau^\zeta} = p_1,$$

ie

$$\theta_0 = -\log(p_0)/\tau^\zeta, \qquad \theta_1 = -\log(p_1).$$

We partition $(0,\tau)$ into m intervals of equal length with boundaries $0 = t_0 < t_1 < t_2 < \ldots < t_m = \tau$ and call the intervals $I_1, I_2, \ldots, I_m$. Consider interval I_k. Let

$$\Delta_{k0} = e^{-\theta_0 t_k^\zeta} - e^{-\theta_0 t_{k+1}^\zeta} \text{ and } \Delta_{k1} = e^{-\theta_1 t_k^\zeta} - e^{-\theta_1 t_{k+1}^\zeta}.$$

The conditional densities, given T is in I_k, are

$$f(t|\tilde{x}=0, I_k) = \frac{\theta_0 \zeta t^{\zeta-1} e^{-\theta_0 t^\zeta}}{\Delta_{k0}}, \quad f(t|\tilde{x}=1) = \frac{\theta_1 \zeta t^{\zeta-1} e^{-\theta_1 t^\zeta}}{\Delta_{k1}}.$$

The conditional survival distribution in the baseline group is

$$S(t|\tilde{x}=0, I_k) = \left\{ e^{-\theta_0 t^\zeta} - e^{-\theta_0 t_{k+1}^\zeta} \right\} / \Delta_{k0}.$$

Now define

$$W = \left\{ e^{-\theta_0 T^\zeta} - e^{-\theta_0 t_{k+1}^\zeta} \right\} / \Delta_{k0} = \left\{ e^{-\theta_0 T^\zeta} - A_{k0} \right\} / \Delta_{k0}, \quad \text{say.}$$

Hence, using the probability integral transform, in the $\tilde{x}=0$ group

$$h(w|\tilde{x}=0, I_k) = 1.$$

The inverse transformation is

$$T = \left(-\frac{1}{\theta_0} \log(\Delta_{k0} W + A_{k0}) \right)^{1/\zeta}$$

and so

$$\begin{aligned} h(w|\tilde{x}=1, I_k) &= \frac{\theta_1 \zeta}{\Delta_{k1}} \left(-\frac{1}{\theta_0} \log(\Delta_{k0} w + A_{k0}) \right)^{(1-1/\zeta)} (\Delta_{k0} w + A_{k0})^{\theta_1/\theta_0} \\ &\quad \times \frac{(\Delta_{k0})}{\zeta \theta_0} \left(-\frac{1}{\theta_0} \log(\Delta_{k0} w + A_{k0}) \right)^{(-1+1/\zeta)} (\Delta_{k0} w + A_{k0})^{-1} \\ &= \frac{\theta_1 \Delta_{k0}}{\theta_0 \Delta_{k1}} (\Delta_{k0} w + A_{k0})^{-1+\theta_1/\theta_0}. \end{aligned}$$

For the curvature we need

$$h'(w|\tilde{x}=1,I_k) = \Delta_{k0}\left(\frac{\theta_1}{\theta_0}-1\right)\frac{\theta_1\Delta_{k0}}{\theta_0\Delta_{k1}}\left(\Delta_{k0}w+A_{k0}\right)^{-2+\theta_1/\theta_0} = B_k\left(\Delta_{k0}w+A_{k0}\right)^{-2+\theta_1/\theta_0},$$

say. And so

$$\int_0^1 \left(h'(w|\tilde{x}=1,I_k)\right)^2 dw = \frac{B_k^2}{\Delta_{k0}\left(2\theta_1/\theta_0-3\right)}\left[\left(\Delta_{k0}+A_{k0}\right)^{-3+2\theta_1/\theta_0} - A_{k0}^{-3+2\theta_1/\theta_0}\right].$$

Tables 4 and 5 show the mean and maximum curvature values over the m intervals for Weibull distributions chosen such that $S(\tau|\tilde{x}=0)=0.7$ and $S(\tau|\tilde{x}=1)=0.2$ (which are the same as in Figure 1), and for various ζ and m. Larger curvature values can occur for other parameter choices, though for any set up that we believe can be considered realistic the curvature is always less than about three, and often substantially lower.

Note that $h(w|\tilde{x})$ is always of the form

$$h(w|\tilde{x}) = \frac{r(a_1-a_2)^r}{(a_1^r-a_2^r)}\left(w+\frac{a_2}{a_1-a_2}\right)^{r-1},$$

where

$$r=\theta_1/\theta_0, \quad a_1=e^{-\theta_0 t_k^\zeta}, \quad a_2=e^{-\theta_0 t_{k+1}^\zeta}.$$

If we set $\psi = 1-a_2/a_1$ then these are all of the form

$$h(w|\tilde{x}) = \frac{r\psi}{(1-(1-\psi)^r)}\left(\psi w+1-\psi\right)^{r-1}.$$

This is the distribution chosen for our efficiency analysis.

	Shape ζ					
	0.333	0.5	0.75	1	1.5	3
$m = 2$	0.525	0.458	0.405	0.392	0.425	0.609
3	0.276	0.221	0.183	0.174	0.193	0.294
4	0.174	0.132	0.104	0.098	0.109	0.170
5	0.122	0.088	0.067	0.063	0.070	0.110
6	0.091	0.063	0.047	0.044	0.049	0.077
7	0.071	0.047	0.034	0.032	0.036	0.057
8	0.057	0.037	0.026	0.025	0.027	0.044
9	0.047	0.030	0.021	0.019	0.022	0.035
10	0.040	0.025	0.017	0.016	0.018	0.028

Table 4: Mean curvatures

	Shape ζ					
	0.333	0.5	0.75	1	1.5	3
$m = 2$	0.983	0.781	0.553	0.392	0.653	1.193
3	0.751	0.522	0.302	0.174	0.325	0.774
4	0.621	0.392	0.196	0.098	0.193	0.523
5	0.535	0.313	0.140	0.063	0.127	0.373
6	0.474	0.261	0.107	0.044	0.090	0.278
7	0.428	0.224	0.085	0.032	0.067	0.215
8	0.392	0.196	0.069	0.025	0.052	0.171
9	0.362	0.174	0.058	0.019	0.041	0.139
10	0.338	0.157	0.050	0.016	0.034	0.115

Table 5: Maximum curvatures
